# Dynamic biochemical tissue analysis detects functional L-selectin ligands on colon cancer tissues

**DOI:** 10.1371/journal.pone.0173747

**Published:** 2017-03-10

**Authors:** Grady E. Carlson, Eric W. Martin, Venktesh S. Shirure, Ramiro Malgor, Vicente A. Resto, Douglas J. Goetz, Monica M. Burdick

**Affiliations:** 1 Department of Chemical and Biomolecular Engineering, Russ College of Engineering and Technology, Ohio University, Athens, Ohio, United States of America; 2 Biomedical Engineering Program, Russ College of Engineering and Technology, Ohio University, Athens, Ohio, United States of America; 3 Biomedical Sciences, Heritage College of Osteopathic Medicine, Ohio University, Athens, Ohio, United States of America; 4 Department of Otolaryngology, University of Texas-Medical Branch, Galveston, Texas, United States of America; 5 Edison Biotechnology Institute, Ohio University, Athens, Ohio, United States of America; Ludwig-Maximilians-Universitat Munchen, GERMANY

## Abstract

A growing body of evidence suggests that L-selectin ligands presented on circulating tumor cells facilitate metastasis by binding L-selectin presented on leukocytes. Commonly used methods for detecting L-selectin ligands on tissues, e.g., immunostaining, are performed under static, no-flow conditions. However, such analysis does not assay for functional L-selectin ligands, specifically those ligands that promote adhesion under shear flow conditions. Recently our lab developed a method, termed dynamic biochemical tissue analysis (DBTA), to detect functional selectin ligands *in situ* by probing tissues with L-selectin-coated microspheres under hemodynamic flow conditions. In this investigation, DBTA was used to probe human colon tissues for L-selectin ligand activity. The detection of L-selectin ligands using DBTA was highly specific. Furthermore, DBTA reproducibly detected functional L-selectin ligands on diseased, e.g., cancerous or inflamed, tissues but not on noncancerous tissues. In addition, DBTA revealed a heterogeneous distribution of functional L-selectin ligands on colon cancer tissues. Most notably, detection of L-selectin ligands by immunostaining using HECA-452 antibody only partially correlated with functional L-selectin ligands detected by DBTA. In summation, the results of this study demonstrate that DBTA detects functional selectin ligands to provide a unique characterization of pathological tissue.

## Introduction

The overexpression of sialofucosylated glycans on colon cancer cells is linked with poor prognosis [1]. Additionally, specific sialofucosylated carbohydrate decorations, e.g., sialyl Lewis x (sLe^x^), have been implicated as adhesion molecules that promote colon cancer metastasis [2]. Mounting evidence suggests that during metastasis sialofucosylated glycans expressed on circulating tumor cells (CTCs), which intravasate into blood from a primary tumor, mediate adhesion with selectin molecules expressed by hematopoietic cells in the blood stream. For example, CTCs expressing L-selectin ligands can bind L-selectin on leukocytes. CTC-leukocyte complexes may adhere to activated endothelial cells that line blood vessel walls and extravasate into secondary organs to form metastatic colonies [3–5].

L-selectin/ligand mediated interactions that are force dependent occur after a threshold level of shear force is provided by hemodynamic flow [6–10]. Under flow conditions, L-selectin/ligand bonds obey catch-slip kinetics that are governed by an intrinsic association constant that dictates the rate of bond formation and a force-dependent dissociation constant that controls the lifetime of the bonds [11–16]. These kinetics result in cell rolling, which is the hallmark of L-selectin/ligand interaction. Rolling interactions occur after cells in flow loosely make contact (i.e., initially tether) with substrates due to L-selectin/ligand interactions and allow the cells to move in the direction of flow while remaining bound to the substrates [17]. Forces imparted on CTC-leukocyte complexes by shear flow are opposed by tensile forces generated by L-selectin/ligand interactions that facilitate CTC-leukocyte rolling on endothelial cells as a preliminary step for extravasation into tissues underlying the endothelium [18].

Detection of L-selectin ligands *in situ* in pathological tissues is traditionally achieved using histological techniques such as immunostaining, e.g., immunohistochemistry (IHC) or immunofluorescence (IF). In these assays, target molecules on tissues are detected with primary constructs, e.g., monoclonal antibodies (mAbs), and secondary conjugated probes under equilibrium “static” conditions. The fundamental reason for using mAbs is that they bind their target via high affinity bonds, which are characterized by relatively low equilibrium dissociation constants (K ≈ 1 x 10^−10^ M) [19–21]. However, due to the plethora of glycoconjugates that can serve as L-selectin ligands, using glycan-specific mAbs to probe for each of these ligands is not pragmatic [22–29]. As an alternative to mAbs, recombinant constructs of L-selectin are used to detect L-selectin ligands in IF. However, L-selectin maintains relatively low affinity bonds with L-selectin ligands in IF assays (K ≈ 1 x 10^−5^ M) [30, 31], making it poorly suited for antigen detection.

Glycoconjugates can be detected in IF, but IF cannot be used to evaluate whether the these glycoconjugates are functional L-selectin ligands, i.e., ligands that bind L-selectin to mediate cell adhesion. While methods of detecting functional selectin ligands on cell lines are well established, e.g., parallel plate laminar flow assays [18, 32], currently the only method available for detecting functional L-selectin ligands on pathological tissues using shear flow is the (rotatation-based) Stamper-Woodruff assay [33]. However, the Stamper-Woodruff assay does not provide highly specific means for controlling how cells or particles interact with the tissue [34]. A method of analysis that engenders forces equivalent to those generated by hemodynamic flow and simultaneously assays for sialofucosylated glycans capable of binding L-selectin is needed in order to definitively detect functional L-selectin ligands.

In this investigation we studied the expression of functional L-selectin ligands *in situ* on colon tissues using dynamic biochemical tissue analysis (DBTA), a technique that introduces controllable flow-generated forces to address shortcomings in ligand detection using traditional tissue analysis methods. Due to the threshold level of fluid shear required for observation of functional L-selectin/ligand activity, we hypothesized that functional L-selectin ligands detected in DBTA would be distinct from L-selectin ligands detected in immunofluorescence (IF). To test this hypothesis we assayed for functional L-selectin ligands that mediated the attachment of L-selectin microspheres to tissues using DBTA, and compared these results with L-selectin ligands detected using IF. Sensitivity and repeatability of the DBTA assay were quantitatively assessed using serial sections of tissue taken from multiple types of colon tissues, including noncancerous and cancerous tissues.

## Methods

### Proteins and antibodies

Purified monoclonal antibody (mAb) HECA-452, which recognizes sialofucosylated moieties found on selectin ligands [26, 27], rat IgMκ isotype control, unconjugated and phycoerythrin (PE) labeled mouse anti-human CD62L mAb (DREG-56) recognizing the lectin binding domain of L-selectin, as well as unconjugated and PE labeled mouse IgGκ isotype control were all purchased from BD Biosciences (San Jose, CA). Immunoglobulin from human placenta (hIgG_1_) was purchased from Sigma (St. Louis, MO). Secondary antibodies, including goat anti-human IgG Alexa Fluor 568 and goat anti-rat IgM Alexa Fluor 647, were obtained from Life Technologies (Carlsbad, CA). Recombinant human L-selectin-hFc chimeric protein (L-selectin) was purchased from R&D Systems (Minneapolis, MN).

### Tissue and cell preparation

Anonymous de-identified formalin-fixed paraffin embedded (FFPE) human tissues of colon origin [signet ring cell carcinoma (SRCC), mucinous adenocarcinoma (MC), papillary adenocarcinoma (PC), and noncancerous (NC) tissues] were purchased from US Biomax, Inc. (Rockville, MD). FFPE tissues were sectioned to a thickness of 5.0 μm on a Leica RM2135 Microtome (Wetzlar, Germany) and mounted on 75 mm x 50 mm microscope slides. Tissue microarrays (TMAs) containing two serial sections of colonic tissues of different types of disease were purchased from US Biomax, Inc.

Ls174T cells were obtained from the American Type Culture Collection (Manassas, VA) and cultured in minimal essential medium supplemented with 10% fetal bovine serum (FBS), 1% penicillin-streptomycin, 1% non-essential amino acids, and 1% sodium pyruvate. Tissue cores consisting of Ls174T cells were prepared as previously described [35]. Briefly, frozen sections were prepared by embedding pelleted Ls174T cells in OCT cryomatrix, and cryosectioning to 5.0 μm. Next, the section was cytofuged to ensure adequate section adhesion to the microscope slide. To prepare FFPE tissue cores, harvested Ls174T cells were pelleted and fixed in 10% neutral buffered formalin for 24 hours at room temperature. The tissue core was then washed in with DPBS+ and dehydrated using a series of graded alcohols and xylenes. Following paraffin wax embedding, Ls174T cores were sectioned to a thickness of 5.0 μm.

Prior to DBTA or IF, FFPE tissue sections were deparaffinized and hydrated using xylenes and graded alcohols (Fisher Scientific Company, Pittsburgh, PA) [36]. Frozen sections were washed three times with Dulbecco’s phosphate buffered solution with calcium and magnesium (DPBS+) to remove the OCT cryomatrix.

### Hematoxylin and eosin staining

Tissues were stained with hematoxylin and eosin as previously described [37]. Briefly, after tissues were deparaffinized and hydrated, they were washed with deionized water and submersed in hematoxylin (Harleco, Gibbstown, NJ) for 20 seconds. Next, tissues were washed with tap water and 70% ethanol, before being submersed in eosin for 30 seconds. After staining, tissues were dehydrated using graded alcohols and xylenes. Tissues were mounted using Permount (Fisher Scientific).

### Immunofluorescence microscopy

The protocol for immunofluorescence was modified from an existing procedure [38]. Following deparafinization and hydration, serial sections of colon tissues were incubated in 1% FBS / 1% bovine serum albumin (BSA) / DPBS+ for one hour. Tissues were incubated in primary labeling solutions of either L-selectin, HECA-452 mAb, hIgG isotype control, or rIgM isotype control (10 μg/ml) for one hour at room temperature. After three washes in DPBS+, secondary antibodies goat anti-rat IgM Alexa Fluor 647 or goat anti-human IgG Alexa Fluor 568, were diluted to 5.0 μg/mL in assay buffer (1% BSA / DPBS+) and applied to tissue for one hour at room temperature. Tissues were then washed twice with DPBS+, and mounted in Prolong Gold mounting medium (Life Technologies) in preparation for imaging. A Leica DMI6000B inverted microscope equipped with a CCD camera was used to capture images of immunolabeled tissue sections imaged using a 10x objective. To control for signal detected from autofluorescence, imaging conditions were optimized using tissues labeled with isotype controls. Subsequently, tissues of matching pathologies that were labeled with specific primary mAbs or protein constructs were imaged using the optimized conditions.

### Microsphere preparation

The procedure for microsphere preparation was modified from an existing protocol [39]. Protein A coated and polystyrene microspheres measuring 10 μm in diameter, were purchased from Bangs Laboratories (Fishers, IN). L-selectin or hIgG proteins were saturated on the surfaces of the microspheres by first incubating the microspheres in 1% BSA / DPBS+ for 30 minutes at room temperature; subsequently microspheres were incubated in tris balanced saline (TBS, pH 8.2) supplemented with L-selectin or hIgG for 30 minutes at room temperature. Following the incubation in L-selectin or hIgG, microspheres were blocked in 1% BSA / DPBS+ for one hour at room temperature and stored at 4^°^C.

Microspheres were characterized using flow cytometry as previously described [40] to determine the incubation concentration at which the microspheres were saturated with L-selectin, such that the microspheres presented the maximum level of L-selectin allowed by their binding capacity (S1 Fig). L-selectin and hIgG microspheres were washed with 0.1% BSA / DBPS+ and incubated with fluorophore conjugated antibodies for 30 minutes at room temperature using a one-step protocol. Subsequently, L-selectin and hIgG microspheres were washed in DBPS+ and analyzed using a FACSAria Special Order Research Product flow cytometer/sorter (BD Biosciences).

### Dynamic biochemical tissue analysis

Tissue sections were washed in DPBS+ and then blocked in 1% FBS / 1% BSA / DPBS+. In experiments assessing the contribution of sialylated molecules to functional L-selectin ligand activity, tissue sections were treated with broadly active sialidase (neuraminidase from *V*. *cholera*) for 30 minutes at 37^°^C. To assay tissues using DBTA, a parallel plate flow chamber was vacuum sealed over tissue section slides, and the assembly was mounted on a Leica DMI6000B inverted microscope equipped with a CCD camera. L-selectin microspheres and hIgG microspheres were separately suspended in a 10 ml reservoir syringe and delivered to the parallel plate flow chamber in 1% BSA / DPBS+ at flow rates calculated to provide the target wall shear stress (S2 Fig, Glycotech, Rockville, MD). The microspheres were delivered to the surface of the tissue at a constant flux using equations derived specifically to describe particle delivery in the parallel plate flow chamber [32]. Microsphere attachment was quantified from recorded videos of DBTA. The initial attachment of microspheres to tissues was termed initial tethering [41]. An interacting microsphere was defined as any microspheres that maintained contact with tissue surface for at least 2 seconds [42]. Rolling microspheres were bound to tissue while simultaneously moving in the direction of flow for 10 seconds while maintaining velocities that were at least 10 fold less than the average velocities of microspheres in the free fluid stream, as previously described [18].

Independent experiments were performed on serial sections of tissues. Each independent DBTA experiment consisted of technical replicate trials: three trials with L-selectin microspheres and three trials with hIgG microspheres. Between each trial microspheres were removed from the system. In certain experiments, L-selectin microspheres were blocked with 10μg/ml anti-human CD62L mAb (DREG-56) or mIgG isotype control for 30 minutes at room temperature.

### Calculation of the rolling velocities of L-selectin microspheres

In offline analysis of DBTA, rolling velocities were evaluated using the MTrackJ tracking software plugin in ImageJ [43], which calculated rolling velocities by dividing the distance that microspheres traveled after three recorded frames by the time elapsed between the first and the third frame (0.33 seconds).

### Statistics

One factor ANOVA was used to assess if the average rolling velocities of L-selectin microspheres were significantly different between the different levels of wall shear stress employed in DBTA. If rolling velocities exhibited significant differences, a Tukey’s multiple comparison test was employed. Two factor ANOVA with repetition was performed to assess if the rolling velocities, number of initial tethering events, number of interactions, or number of rolling L-selectin microspheres and hIgG microspheres were significantly different on serial sections of colon cancer tissues and noncancerous colon tissues at the assigned levels of wall shear stress used in DBTA.

## Results

### Colon cancer tissues present L-selectin ligands and sialofucosylated glycoconjugates detectable by IF

**Fig 1:**
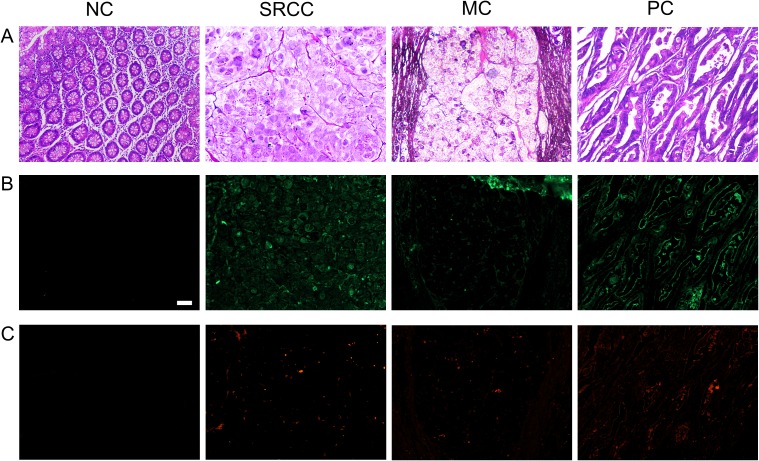
Colon cancer tissues express purported L-selectin ligands. Noncancerous (NC), signet ring cell carcinoma (SRCC), mucinous adenocarcinoma (MC), and papillary adenocarcinoma (PC) colon tissues were assayed using **(A)** hematoxylin and eosin to assess the histological classification of each tissue and **(B)** HECA-452 mAb to detect sialofucosylated carbohydrate decorations using IF, or **(C)** L-selectin to detect L-selectin ligands using IF. Tissue sections were cut from FFPE tissue blocks and data are representative of n = 3 independent experiments, as described in Methods. Scale bar = 100 μm. Images were acquired using a 10x objective.

In this study the pathological classifications of noncancerous tissue (NC), signet ring cell carcinoma (SRCC), mucinous adenocarcinoma (MC), and papillary adenocarcinoma (PC) colon tissues were verified using hematoxylin and eosin staining (Fig 1A). HECA-452 antigens (green, Fig 1B) and ligands detected by L-selectin (red, Fig 1C) were detected on colon cancer tissues using IF, yet no signal was detected on the noncancerous tissue (control). Furthermore, tissues labeled with isotype controls had low (i.e., nearly undetectable) levels of staining (S3 Fig), indicating that signals detected in IF using L-selectin or HECA-452 were target-specific. Additionally, greater levels of expression of purported L-selectin ligands were detected on tissues assayed with HECA-452 compared to tissues assayed with L-selectin. This result was anticipated because the HECA-452 mAb was expected to bind purported L-selectin ligands with greater affinity than the L-selectin protein in IF assays [19, 20, 30, 31].

### Dynamic biochemical tissue analysis allows detection of functional L-selectin ligands that distinguish cancerous and noncancerous tissues

**Fig 2:**
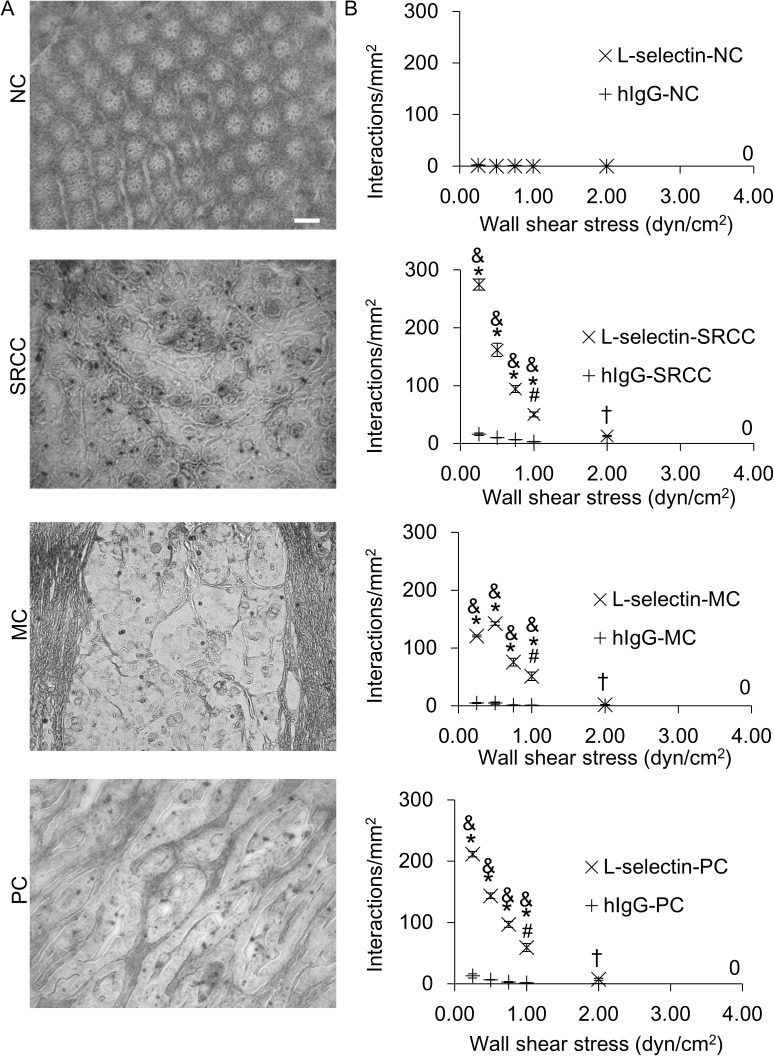
Functional L-selectin ligands on colon cancer tissues that mediate L-selectin/ligand interactions with L-selectin microspheres were detected using DBTA. **(A)** Images of DBTA of colon cancer tissues were captured in real-time using an inverted microscope equipped with a CCD camera, as L-selectin microspheres interacted with L-selectin ligands on cancerous tissues. **(B)** A significantly greater number of L-selectin microspheres interacted with L-selectin ligands on colon cancer tissues compared to noncancerous (NC) tissues (&*P<*0.05) or human hIgG microspheres (*P<0.05). The number of L-selectin microsphere interactions was significantly reduced at 1.0 dyn/cm^2^, relative to the number of interactions that occurred between 0.25–0.50 dyn/cm^2^ (#*P*<0.05). Additionally, signifcantly fewer L-selectin microsphere interactions occurred at wall shear stress of 2.0 dyn/cm^2^ compared to the number of L-selectin microsphere interactions that occurred at wall shear stresses of 0.25–1.0 dyn/cm^2^ (†*P*<0.05). Microspheres were perfused over tissues at a constant flux. Tissue sections were cut from FFPE tissue blocks and data are mean ± SEM for n = 3 independent experiments, as described in Methods. Scale bar = 100 μm. Images were acquired using a 10x objective.

To determine if L-selectin ligands were functional under hydrodynamic shear forces, colon tissues were assayed using dynamic biochemical tissue analysis (DBTA, Fig 2A) with microspheres conjugated with L-selectin (S1 Movie) or hIgG (negative control, S2 Movie). The number of L-selectin microsphere interactions were significantly greater than the number of hIgG microsphere interactions (Fig 2B), demonstrating the specificity of the L-selectin/ligand interaction. To further investigate the specificity of the L-selectin/ligand interaction, L-selectin microspheres were also perfused over tissues in the presence of ethylenediaminetetraacetic acid (EDTA, S3 Movie), which chelates divalent cations, or over tissues pre-treated with sialidase (S4 Movie), which cleaves sialic acids that decorate the L-selectin binding domain of certain L-selectin ligands. In each case the number of L-selectin microsphere interactions on EDTA or sialidase-treated tissues was significantly less than the number of L-selectin microsphere interactions on untreated colon cancer tissues, substantiating that L-selectin/ligand interactions observed in DBTA were specific (S4A Fig). These data are consistent with reports showing that L-selectin/ligand interactions are calcium dependent and that L-selectin ligands are decorated with sialofucosylated carbohydrates [29, 44–47]. Furthermore, anti-L-selectin mAb blockade [42, 43] of L-selectin microspheres nearly abolished L-selectin microsphere interactions with SRCC tissue, yet the the mIgG isotype control had no effect on L-selectin microsphere tethering (S4B Fig).

To explore how varying the forces that are generated in hydrodynamic flow during DBTA affect the results of the assay, microspheres were perfused over tissues at a constant flux using a range of wall shear stresses (0.25–4.00 dyn/cm^2^, Fig 2B). Increasing the value of wall shear stress significantly reduced the number of L-selectin microsphere, and at 4 dyn/cm^2^ L-selectin microspheres did not attach to tissues. Increasing the value of wall shear stress significantly reduced the number of L-selectin microsphere interactions, and L-selectin microsphere interactions did not occur 4 dyn/cm^2^. These data are consistent with published reports showing that the dynamics of L-selectin/ligand interactions are influenced by wall shear stress generated by shear flow [6, 8, 48, 49]. Importantly, Fig 2B clearly reveals that L-selectin microspheres distinguished cancerous and noncancerous tissues. That is, the number of L-selectin microsphere interactions on colon cancer tissues was significantly greater than the number of L-selectin microsphere interactions on noncancerous tissues.

**Fig 3:**
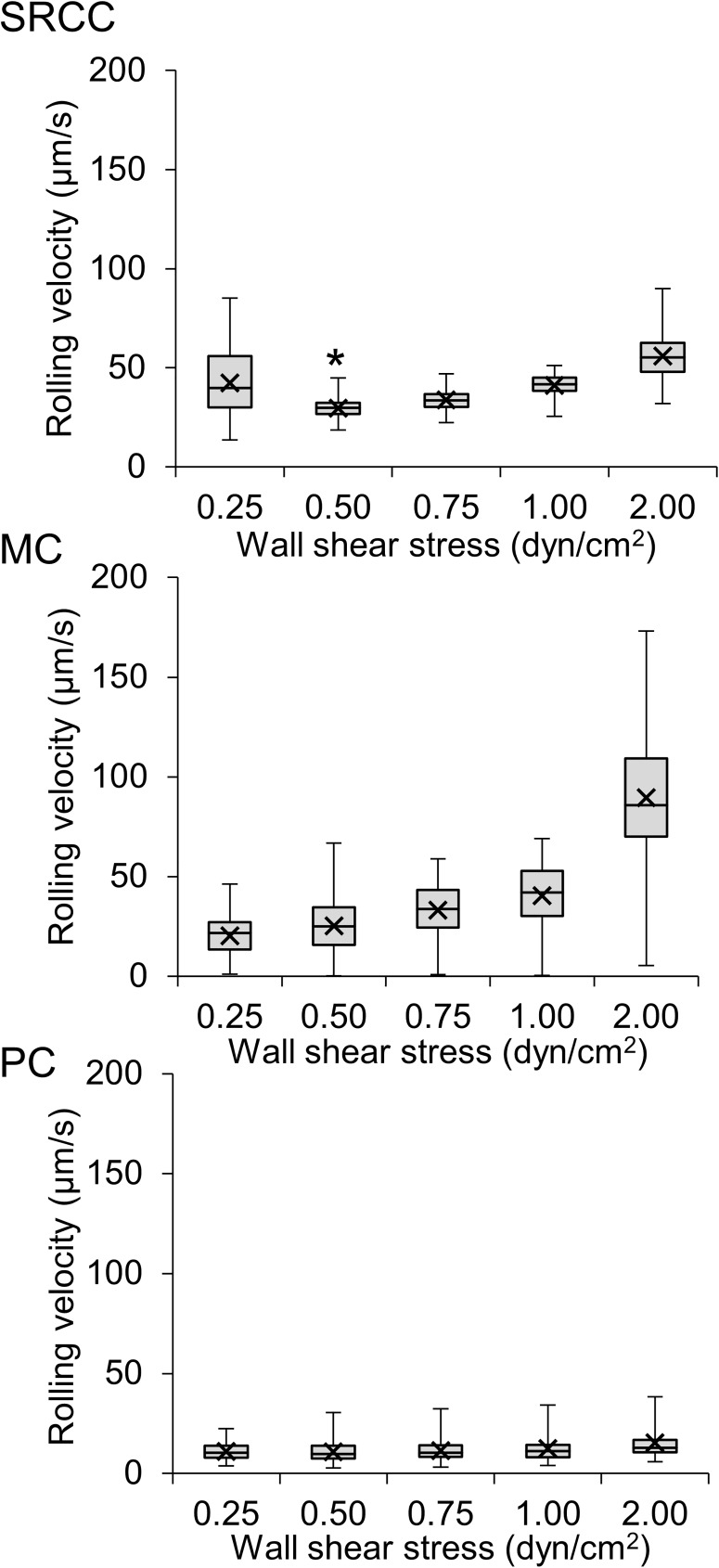
Rolling velocity profiles of L-selectin microspheres distinguish tissues assayed in DBTA. The mean value (X) of the rolling velocities of the L-selectin microspheres that rolled on colon cancer tissues in DBTA was measured across multiple levels of wall shear stress and demonstrated the force dependence of L-selectin/ligand mediated rolling. Rolling velocities of L-selectin microspheres achieved a minimum on SRCC tissue at 0.50 dyn/cm^2^ (*P<0.05). Tissue sections were cut from FFPE tissue blocks and data are mean ± SEM for n = 3 independent experiments, as described in Methods. A total of 180 microspheres were analyzed for each wall shear stress. Box and whisker plots illustrate the mean (X) rolling velocities of L-selectin microspheres, the median (middle line), the upper quartile and lower quartile values (edges of box), and the range of the microspheres’ rolling velocities (error bars).

To further investigate how changes in wall shear stress affected the detection of functional L-selectin ligands using DBTA, rolling velocities of L-selectin microspheres that attached to tissues were calculated and analyzed at multiple levels of wall shear stress. SRCC colon tissues supported rolling of L-selectin microspheres at wall shear stresses between 0.25–2.00 dyn/cm^2^. At 0.50 dyn/cm^2^ the average rolling velocities of these L-selectin microspheres was at a minimum (Fig 3). This decrease in the rolling velocities of L-selectin microspheres that occurred as the wall shear stress was increased from 0.25 dyn/cm^2^ to 0.50 dyn/cm^2^ suggests that the duration of the L-selectin/ligand bond increased in response to an increase in shear force (wall shear stress). This force dependent behavior is characteristic of catch bonds [12]. On the other hand, the increase in microsphere rolling velocity beyond 0.50 dyn/cm^2^ shear stress, reflects slip bond behavior. Thus, under the tested conditions, the L-selectin ligands expressed on SRCC tissue appear to have kinetic properties that allow a catch-to-slip bond transition [16] and shear-enhanced L-selectin/ligand mediated rolling. Furthermore, although MC and PC tissues supported rolling of L-selectin microspheres, the rolling velocities of L-selectin microspheres on SRCC, MC, and PC tissues were different and indicate that each type of tissue expressed different L-selectin ligands and/or had a heterogeneous presentation of L-selectin ligands. For example, L-selectin ligands expessed on MC or PC tissues did not appear to undergo a catch-to-slip bond transition under the assayed conditions (Fig 3). Additionally, the average rolling velocities of L-selectin microspheres on PC tissues were lower than the average rolling velocities of L-selectin microspheres on SRCC or MC tissues and were independent of the magnitude of the wall shear stress (Fig 3).

### The detection of functional L-selectin ligands on colon cancer tissues using DBTA is distinct from the detection of L-selectin ligands using immunofluorescence

**Fig 4:**
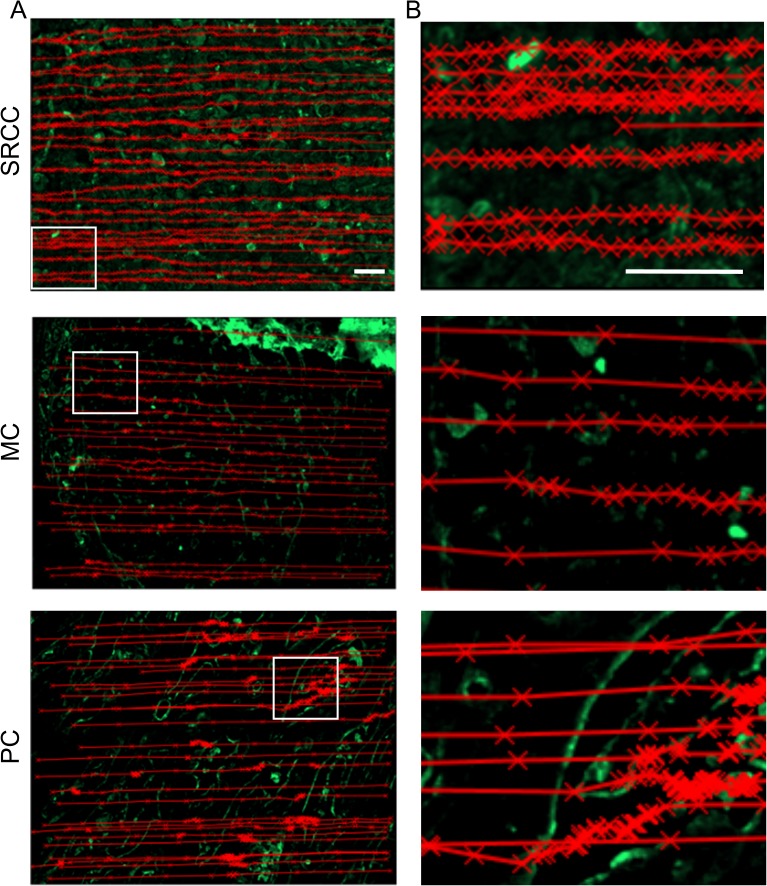
DBTA provides a distinct characterization of pathological tissues by detecting functional L-selectin ligands. **(A)** L-selectin microspheres were tracked using ImageJ software and an MtrackJ plugin as they tethered and rolled on SRCC, MC, and PC tissues in DBTA. The recorded microspheres tracks (-X-) were overlaid onto images from the same colon cancer tissues that were subsequently assayed with IF using HECA-452 mAb (green), which detects potential L-selectin ligands. Comparison of the microsphere tracks and the L-selectin ligands detected using IF demonstrated that the detection of functional L-selectin ligands in DBTA was distinct from the detection of selectin ligands using IF. **(B)** Enlarged regions of interest outlined in white in (A) show that as L-selectin microspheres were tracked rolling on tissue, their tracks became clustered as more avid and/or higher afinity L-selectin/ligand bonds caused the microspheres to roll with a lower velocity. Positioning of the track overlays from the phase contrast movie to the IF image was accomplished by coordinate mapping of the motorized microscope stage and imaging software. Tissue sections were cut from FFPE tissue blocks and data are representative of n = 3 independent experiments, as described in Methods. Scale bars = 100 μm. Images were acquired using a 10x objective.

To compare the results of immunostaining and DBTA, images of tissues assayed in IF using HECA-452 mAb (which provided enhanced detection of potential functional L-selectin ligands in IF compared to L-selectin proteins, Fig 1B and 1C) were overlaid with the tracks of L-selectin microspheres that rolled on tissues in DBTA (Fig 4A). Analysis of these overlays revealed that the IF signals only partially correlated with microsphere tracks (-X-), which became clustered in regions where microspheres rolled on tissues during DBTA (Fig 4B). Importantly, some regions of tissue that promoted microsphere rolling in DBTA did not present detectable signals in IF. The detection of functional L-selectin ligands in DBTA that occurred in regions of tissues that lacked detectable IF signals demonstrates that force-dependent L-selectin/ligand interactions comprise a distinct measurable dimension for tissue characterization that is unique to DBTA.

### DBTA reveals a heterogeneous distribution of functional L-selectin ligands on colon cancer tissues

Analysis of the rolling of L-selectin microspheres on colon cancer tissues assayed in DBTA revealed a heterogeneous presentation of functional L-selectin ligands on colon cancer tissues. Microspheres were tracked as they rolled on tissues using a tracer (X) to mark their coordinates on the tissue in 0.33 second intervals (three frames of video). Tracers indicating the position of the microspheres were connected with a line to track the path of the microspheres. Closely spaced tracers indicate where microspheres rolled with relatively slow velocities and potentially indicate regions of tissue that present a high density of and/or higher affinity L-selectin ligands (Fig 4B). In contrast, tracks generated from microspheres that rolled with relatively large velocities consisted of tracers that were spaced further apart suggesting that these microspheres rolled in regions with low densities of L-selectin ligands (Fig 4B). Examination of the tracks from L-selectin microspheres that rolled on MC and PC tissues in DBTA revealed region-specific rolling of L-selectin microspheres (regions in which tracks are tightly clustered) and thus heterogeneity in the presentation of functional L-selectin ligands on these tissues (Fig 4A and 4B). In contrast, L-selectin microspheres rolled across most of the SRCC tissue (Fig 4A).

**Fig 5:**
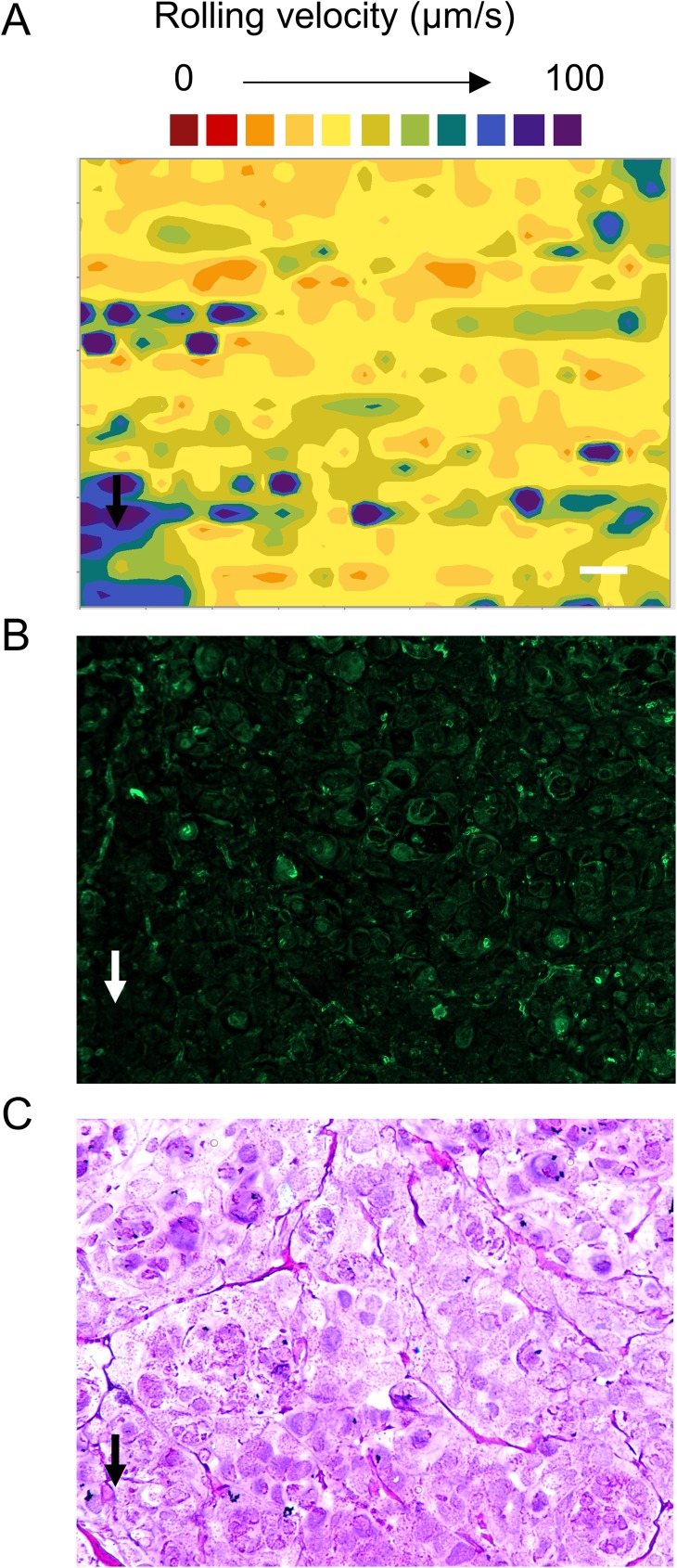
The functional L-selectin ligands detected on SRCC tissue using DBTA exhibited a heterogeneous distribution. **(A)** The heterogeneous distribution of functional L-selectin ligands detected on SRCC tissue using DBTA was examined using a color coded contour plot of the magnitude of the velocities of L-selectin microspheres that rolled on SRCC tissue. The colors used to construct the contour plot are proportional to the rolling velocities of the L-selectin microspheres, e.g., shades of red indicate relatively low magnitude velocities and shades of violet indicate relatively large magnitude velocities. The black arrow (bottom left) indicates a representative region of tissue in which microspheres rolled at high velocity. (B) IF image of signals detected from fluorophore conjugated secondary antibodies that bound HECA-452 primary antibodies on SRCC tissues. The white arrow (bottom left) indicates the region shown in (A). L-selectin microspheres were perfused over tissues at a wall shear stress of 0.50 dyn/cm^2^. Tissue sections were cut from FFPE tissue blocks and data are representative of n = 3 independent experiments, as described in Methods. The contour map was generated from the tracks of twenty L-selectin microspheres. Scale bar = 100μm. Images were acquired using a 10x objective.

Despite the relatively homogeneous spatial distribution of rolling L-selectin microsphere tracks on SRCC tissue, a contour plot of the rolling velocities revealed localized functional L-selectin ligand activities (Fig 5A). L-selectin microsphere rolling velocities are depicted with spectrum colors. Shades of red indicate low magnitude rolling velocities (high density of L-selectin ligands and/or high affinity L-selectin ligands), and shades of violet designate high magnitude rolling velocities (low density of L-selectin ligands and/or low affinity L-selectin ligands). The nonhomogeneous color distribution in the contour plot (Fig 5A) indicates that the SRCC tissue exhibited a heterogeneous distribution of functional L-selectin ligands. Furthermore, the spatial distribution of localized L-selectin ligand activities shown on the contour plot (Fig 5A) appears distinct from the spatial distribution of L-selectin ligands detected using IF (Fig 5B).

### Serial sections of colon tissues similarly express functional L-selectin ligands

**Fig 6:**
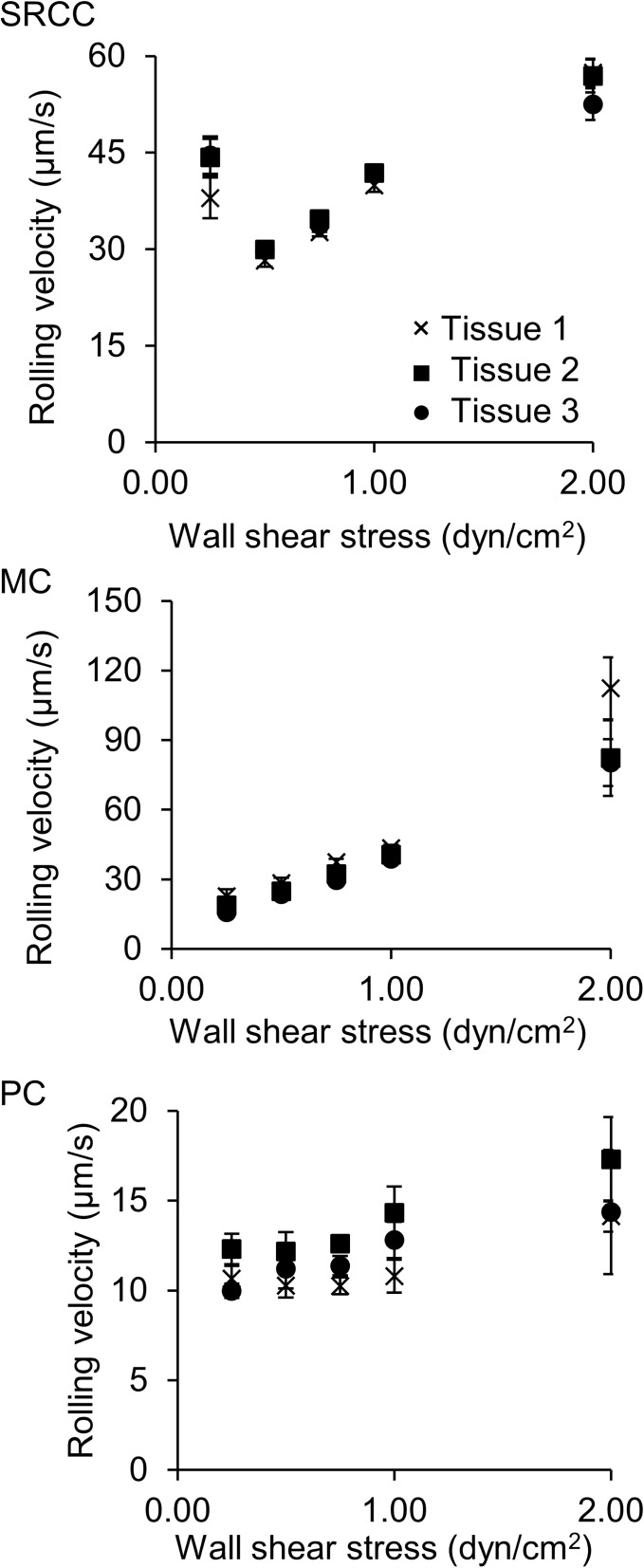
DBTA allows repeatable detection of functional L-selectin ligands on serial sections of colon tissues cut from the same tumor. L-selectin microspheres were perfused over three 5 μm thick serial sections of **(A)** SRCC, **(B)** MC, and **(C)** PC colon cancer tissues in DBTA at wall shear stresses of 0.25–4.00 dyn/cm^2^. The rolling velocities of L-selectin microspheres on serial sections of colon cancer tissues resected from the same origin were not significantly different, indicating that similar results from DBTA can be obtained from serial sections of colon tissues. L-selectin microspheres did not adhere to tissues in DBTA at 4.00 dyn/cm^2^. Tissue sections were cut from FFPE tissue blocks and data are mean ± SEM for n = 3 independent experiments, as described in Methods. A total of 180 microspheres were analyzed for each wall shear stress.

Serial sections of tissues (5 μm thick) were assayed for functional L-selectin ligand expression using DBTA to determine if separate tissue sections from the same colon tissue similarly expressed functional L-selectin ligands, within the approximate diameter of a cell (15 μm, 3 sections). The expression of functional L-selectin ligands on colon tissues was quantified by calculating the average rolling velocities of the L-selectin microspheres. For any tested level of wall shear stress, the average rolling velocities of the L-selectin microspheres on SRCC (Fig 6A), MC (Fig 6B), and PC (Fig 6C) colon tissues were not significantly different between the serial sections for a given type of tissue, indicating that serial tissue sections from the same tissue block similarly expressed functional L-selectin ligands.

### Formalin-fixation preserves functional L-selectin ligands on colon cancer tissues

**Fig 7:**
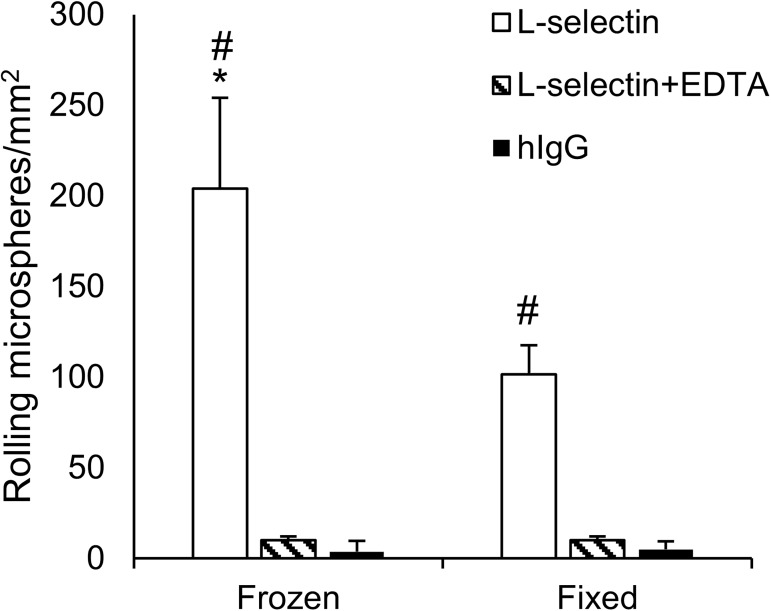
DBTA detects functional L-selectin ligands on frozen and formalin-fixed tissue sections. L-selectin or hIgG microspheres were perfused over frozen and formalin-fixed tissue sections comprised of Ls174T cells at a wall shear stress of 1.25 dyn/cm^2^. Significantly greater numbers of L-selectin microspheres rolled on frozen and formalin-fixed tissue compared to the number of rolling hIgG microspheres or L-selectin microspheres in the presence of EDTA (#P<0.05). The number of L-selectin microspheres that rolled on frozen tissues was significantly greater than the number of L-selectin microspheres that rolled on formalin-fixed tissues (*P<0.05). Data are mean ± SEM for n = 3 independent experiments.

Tissue fixation and processing can modulate antigen detection [50, 51]. To evaluate whether the detection of functional L-selectin ligands in DBTA is modified by the effects of formalin-fixation and tissue processing, DBTA was performed using frozen or FFPE sections from tissue cores comprised of Ls174T colon cancer cells. Significantly greater numbers of L-selectin microspheres rolled on frozen and formalin-fixed tissues compared to the number of hIgG microspheres or L-selectin microspheres in the presence of EDTA, indicating that the rolling of L-selectin microspheres was mediated by calcium-dependent L-selectin/ligand interactions (Fig 7). Additionally, significantly greater numbers of L-selectin microspheres rolled on frozen tissues compared to the number of L-selectin microspheres that rolled on formalin-fixed tissues (Fig 7). Thus neither formalin-fixation nor tissue processing abolished or artificially enhanced the detection of functional L-selectin ligands on colon cancer tissues assayed using DBTA. Formalin-fixation appeared to decrease the number of L-selectin microspheres that rolled on tissues, but reduced antigenicity after formalin-fixation is common [50, 51]. Reduced antigenicity of Ls174T tissue cores due to formalin-fixation was also observed when frozen and formalin-fixed tissues were assayed for HECA-452 antigens using IF, because frozen tissues had greater signal intensities than tissues that were formalin-fixed (S5 Fig). Thus, the observation that fewer L-selectin microspheres rolled on formalin-fixed tissues compared to frozen tissues (Fig 7) indicates that some, but not all, L-selectin ligands may be lost, become undetectable, or are rendered nonfunctional by formalin-fixation.

### Functional L-selectin ligands are expressed at different levels on colon tissues of different histopathological classifications

In order to determine whether DBTA may be used to detect functional L-selectin ligands that are purportedly expressed on primary tumors, metastatic tumors, inflamed tissue, and hyperplastic tissue [52], 51 cases of colon tissues were assayed for functional L-selectin ligands using DBTA. L-selectin microspheres rolled on most pathological tissues, but did not attach to noncancerous (control) colon tissues (Table 1). In most cases, serial sections of tissue demonstrated similar levels of L-selectin ligand activity. However, L-selectin ligands were not present on all of the histological classifications of diseased colon tissues. For example, L-selectin microspheres did not roll on any carcinoid colon tissues, yet L-selectin microspheres tethered and rolled on 83% of metastatic mucinous adenocarcinoma tissues assayed in DBTA. Thus, these data support prior investigations, which show that L-selectin ligands are not ubiquitously overexpressed in all types of cancer [52, 53].

**Table 1:** DBTA detects greater levels of functional L-selectin ligand activity on colon tissues resected from patients with inflammation, hyperplasia, or cancer relative to noncancerous tissues.

Histological classification of patient cases in the colon TMA	L-selectin ligand positive cases of colon cancer (supported rolling)	Total number of cases	Percent L-selectin ligand positive [(L-selectin positive / total number)x100)]
Noncancerous (normal)	0	12	0%
Inflammation	7	17	41%
Hyperplasia	17	21	81%
Mucinous adenocarcinoma	4	8	50%
Signet ring cell carcinoma	5	5	100%
Papillary adenocarcinoma	2	3	67%
Metastatic adenocarcinoma	4	26	15%
Metastatic mucinous adenocarcinoma	10	12	83%
Carcinoid tumor	0	6	0%

## Discussion

A growing body of work supports that the overexpression of selectin ligands on colon cancer cells is linked with poor prognosis. Selectin/ligand interactions may facilitate the adhesion of CTCs with hematopoietic cells and vascular endothelial cells to promote cancer metastasis [1, 53–58]. Specifically, CTCs expressing selectin ligands may bind leukocytes (expressing L-selectin), platelets (expressing P-selectin), and endothelial cells (expressing E-selectin and P-selectin) to facilitate the initial stages of adhesion in the colonization of distal metastases [3, 4, 54, 56, 59–62]. Until now, the ability to detect functional selectin ligands, i.e., ligands that facilitate L-selectin ligand interactions, on tissues under well-controlled conditions has been limited, because functional selectin/ligand binding is facilitated through unique kinetics and tensile forces that are not provided by traditional “static” methods of tissue analysis, e.g., IF.

In this study we investigated the feasibility of a novel method of tissue analysis termed dynamic biochemical tissue analysis (DBTA), to detect functional L-selectin ligands *in situ* on colon tissues. Due to the application of flow, DBTA engenders conditions requisite for detecting functional L-selectin ligands, which have been implicated in CTC trafficking during colon cancer metastasis [4, 13–16, 63]. L-selectin/ligand interactions with kinetics governed by force-dependent dissociation constants increase their lifetime as applied force is increased, until a threshold level of force applied by fluid shear is acheived [10, 12–14, 64, 65]. DBTA of SRCC tissues using L-selectin microspheres revealed the force-dependent nature of the L-selectin/ligand bond as the rolling velocities of L-selectin microspheres reached a minimum as the wall shear stress was increased from 0.25 dyn/cm^2^ to 0.50 dyn/cm^2^ (Fig 3). The observation of this force-dependent adhesion has also been reported for colon cancer cells that formed L-selectin/ligand bonds with peripheral blood mononuclear cells (PBMCs) under shear flow [41]. In that study L-selectin-mediated adhesion between PMBCs and colon cancer cells ceased when the flow was reduced below a threshold level of shear [41].

By assaying pathological tissues for functional selectin ligands using DBTA, which uniquely allows repeatable detection of functional selectin ligands on serial sections of tissues (Fig 6), we revealed a spatially heterogeneous distribution of functional L-selectin ligands *in situ* on FFPE colon cancer tissues (Figs 4 and 5). The utility of DBTA was highlighted by the fact that it can be used to detect functional L-selectin ligands on both frozen and FFPE tissues (Fig 7), which are widely available. However, several factors must be considered in the evaluation of tissue specimens for assaying with DBTA. Tissue fixation, processing, and storage protocols [51, 66] may affect tissue antigenicity and thus the results of DBTA. Other factors such as tissue surface area [64] and amount of connective tissue, invasive leukocytes, or non-parenchymal cells that may or may not express L-selectin ligands should be carefully considered as well (these cells may be identified using H&E staining or immunostaining for specific biomarkers).

Along with standard methods of tissue analysis, such as IF, IHC, and fluorescence *in situ* hybridization (FISH), DBTA may potentially be used as a diagnostic or prognostic tool for cancers in which an overexpression functional selectin ligands is associated with metastasis and poor patient outcomes [53]. DBTA detected functional L-selectin ligands on diseased tissues, yet noncancerous tissue did not exhibit functional L-selectin ligand activity (Fig 2). These results indicate that functional L-selectin ligands are potential biomarkers of colonic disease. Although mouse studies indicate that decreased expression of L-selectin or L-selectin ligands reduces tumor-leukocyte interactions and attenuates cancer metastasis [4], the mechanisms of L-selectin/ligand mediated metastasis in humans remain unclear.

In summary, the results of the investigation herein demonstrate that detection of functional L-selectin ligands on pathological tissues can be accomplished using DBTA. Additionally, some of the functional L-selectin ligands detected using DBTA were found in regions of tissue where L-selectin ligands were not detected using IF, revealing that DBTA provides a unique characterization of pathological tissue. Although additional investigation is needed to elucidate the clinical significance of the different levels of functional L-selectin ligands detected on colon cancer tissues, our findings highlight that DBTA is a feasible, specific, and repeatable assay that can be used alongside other methods of tissue analysis. Ultimately, DBTA may be utilized to detect functional selectin ligands on tissues *in situ*, bolstering efforts to improve diagnosis and treatment of cancers derived from solid tumors.

## Supporting information

S1 Fig Protein A microspheres were conjugated with L-selectin-hFc chimera using saturating concentrations of L-selectin-hFc chimera, which provided a maximum level of L-selectin presentation on protein A microspheres. **(A)** Mean fluorescence intensities of protein A microspheres were analyzed in flow cytometry and increased with increasing incubation concentrations of L-selectin to determine the concentration at which saturating levels were achieved (≥ 12 μg/ml). Data are mean ± SEM for n = 5 independent experiments. **(B)** Flow cytometry histograms of L-selectin microspheres prepared with increasing incubation concentrations of L-selectin. Data are representative of n = 5 independent experiments.
(TIF)

S2 Fig Experimental setup for dynamic biochemical tissue analysis (DBTA). Microspheres are suspended in a reservoir and delivered to a parallel plate flow chamber using a syringe pump. A vacuum seals the flow chamber atop the tissue section (mounted on a microscope slide). Images of real-time microsphere adhesion events are captured using an inverted microscope and a CCD camera, which records the images to a computer for offline analysis.
(TIF)

S3 Fig Signal was nearly undetectable from isotype controls for primary specific IF constructs. The IF analysis of tissues using isotype controls included **(A)** rIgM for HECA-452 and **(B)** hIgG for L-selectin. Imaging conditions for isotype controls were used to control for autofluorescence. Scale bar = 100 μm. Images were acquired using a 10x objective. Tissue sections were cut from FFPE tissue blocks and data are representative of n = 3 independent experiments, as described in Methods.
(TIF)

S4 Fig L-selectin microspheres adhered to colon cancer tissues via L-selectin/ligand interactions. **(A)** Significantly fewer L-selectin microsphere interactions occurred on colon cancer tissues in the presence of EDTA (5mM) or on colon cancer tissues treated with sialidase. Data are mean ± SEM for n = 3 independent experiments, as described in Methods (*P<0.05). **(B)** The initial tethering of L-selectin microspheres to SRCC cancer tissue was significantly decreased (almost to complete blockade) by function blocking anti-Lselectin mAb but not mIgG isotype control. Data are mean ± SEM for n = 3 replicate flow assays on one SRCC tissue section (*P<0.05). Tissue sections were cut from FFPE tissue blocks as described in Methods.
(TIF)

S5 Fig Analysis of IF assays of frozen and formalin-fixed tissues using HECA-452 shows that greater signal intensities were detected on frozen tissues compared to formalin-fixed tissues. Histograms report the fluorescence intensity of image pixels from IF of frozen or fixed Ls174T tissues assayed with HECA-452 mAb or rIgM isotype control. A greater number of pixels had higher HECA-452 fluorescence intensities on frozen tissue than on fixed tissue, yet tissues stained with HECA-452 had greater intensities than the isotype control tissues regardless of the preparation technique. Data shown are, signal intensities collected from a single representative tissue section of frozen or FFPE tissues (corresponding to n = 3 experiments in Fig 7). Scale bar = 100 μm.
(TIF)

S1 Movie **L-selectin microspheres were perfused over (A) SRCC, (B) MC, (C) PC, and (D) NC colon tissues in DBTA.** L-selectin microspheres were tracked (-X-) as they rolled on colon cancer tissues in order to calculate their rolling velocities. L-selectin microspheres did not roll on NC tissues. Movies were acquired using a 10x objective.
(MP4)

S2 Movie **hIgG microspheres were perfused over (A) SRCC, (B) MC, (C) PC, and (D) NC colon tissues in DBTA.** Significantly fewer hIgG microsphere interactions occurred on colon tissues in DBTA relative to the number of L-selectin microsphere interactions, demonstrating that L-selectin microsphere interactions on colon cancer tissues were due to L-selectin/ligand bonds. Movies were acquired using a 10x objective.
(MP4)

S3 Movie L-selectin microspheres did not adhere to colon cancer tissues in the presence of EDTA. L-selectin microspheres ceased to adhere to tissues in the presence of EDTA, which chelates calcium ions requisite for the L-selectin ligand interactions. EDTA abolished L-selectin microsphere adhesion after 18 seconds of video. Movie was acquired using a 10x objective.
(MP4)

S4 Movie Sialidase treatment abolished the adhesion of L-selectin microspheres to colon cancer tissues. After sialidase treatment, L-selectin microspheres ceased to attach to **(A)** SRCC, **(B)** MC, and **(C)** PC colon cancer tissues. Movies were acquired using a 5X objective.
(AVI)

S5 Movie Tracks of L-selectin microspheres on colon cancer tissues were compared to images captured in IF. Two representative L-selectin microspheres were tracked as they rolled on SRCC colon tissue (originally imaged in phase contrast), and tracks were overlaid onto the IF image of the same SRCC tissue that was stained using HECA-452 subsequent to DBTA. Positioning of the track overlays from the phase contrast movie to the IF image was accomplished by coordinate mapping of the motorized microscope stage and imaging software. Movie was acquired using a 10X objective.
(MP4)
