# Supplementary figures and images for: Dynamic biochemical tissue analysis detects functional L-selectin ligands on colon cancer tissues
Source: PLoS One. 2017 Mar 10;12(3):e0173747. doi: 10.1371/journal.pone.0173747 (PMC5345883; doi:10.1371/journal.pone.0173747)

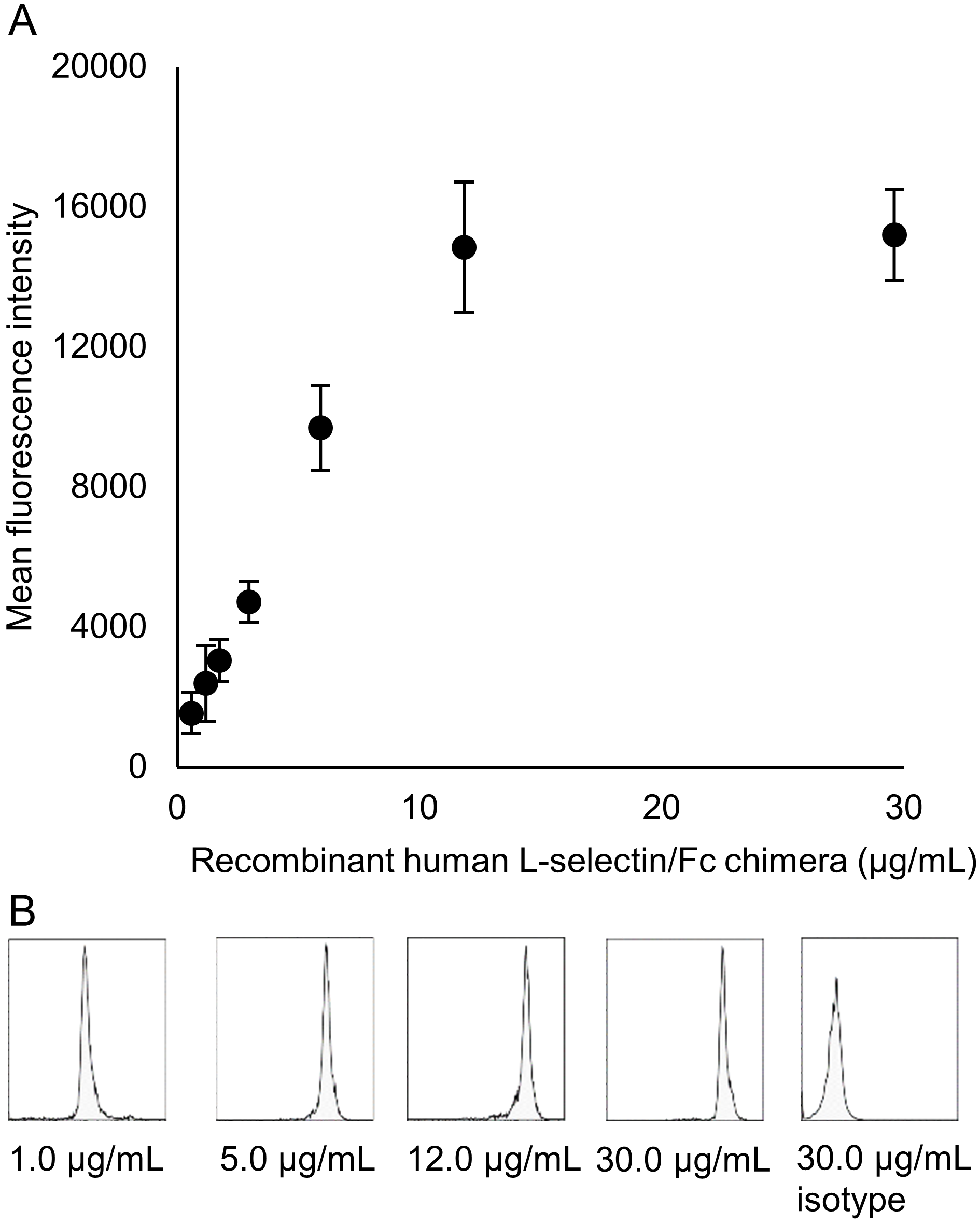

Supplement: S1 Fig — (A) Mean fluorescence intensities of protein A microspheres were analyzed in flow cytometry and increased with increasing incubation concentrations of L-selectin to determine the concentration at which saturating levels were achieved (≥ 12 μg/ml). Data are mean ± SEM for n = 5 independent experiments. (B) Flow cytometry histograms of L-selectin microspheres prepared with increasing incubation concentrations of L-selectin. Data are representative of n = 5 independent experiments. (TIF) [file pone.0173747.s001.tif]

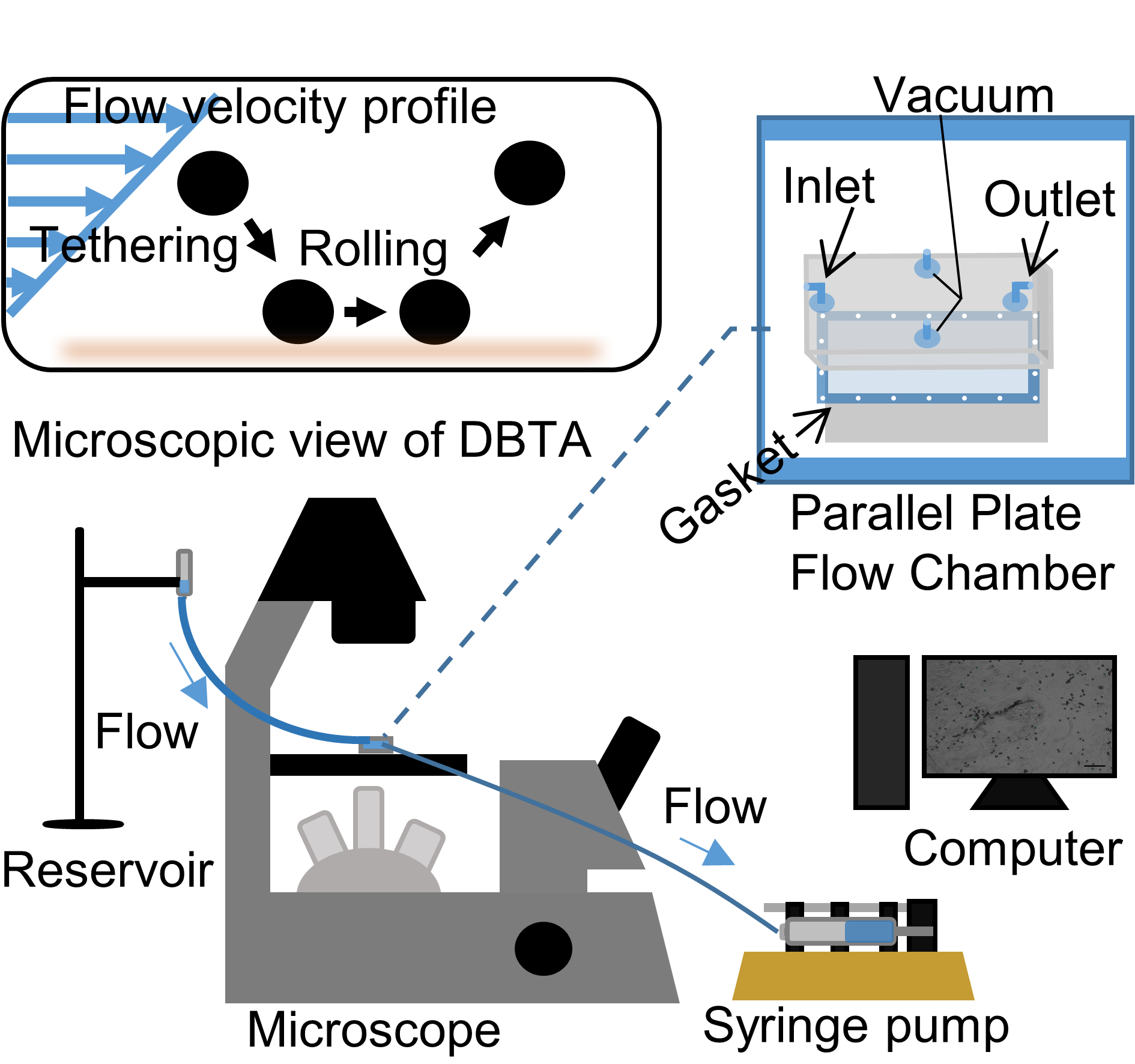

Supplement: S2 Fig — Microspheres are suspended in a reservoir and delivered to a parallel plate flow chamber using a syringe pump. A vacuum seals the flow chamber atop the tissue section (mounted on a microscope slide). Images of real-time microsphere adhesion events are captured using an inverted microscope and a CCD camera, which records the images to a computer for offline analysis. (TIF) [file pone.0173747.s002.TIF]

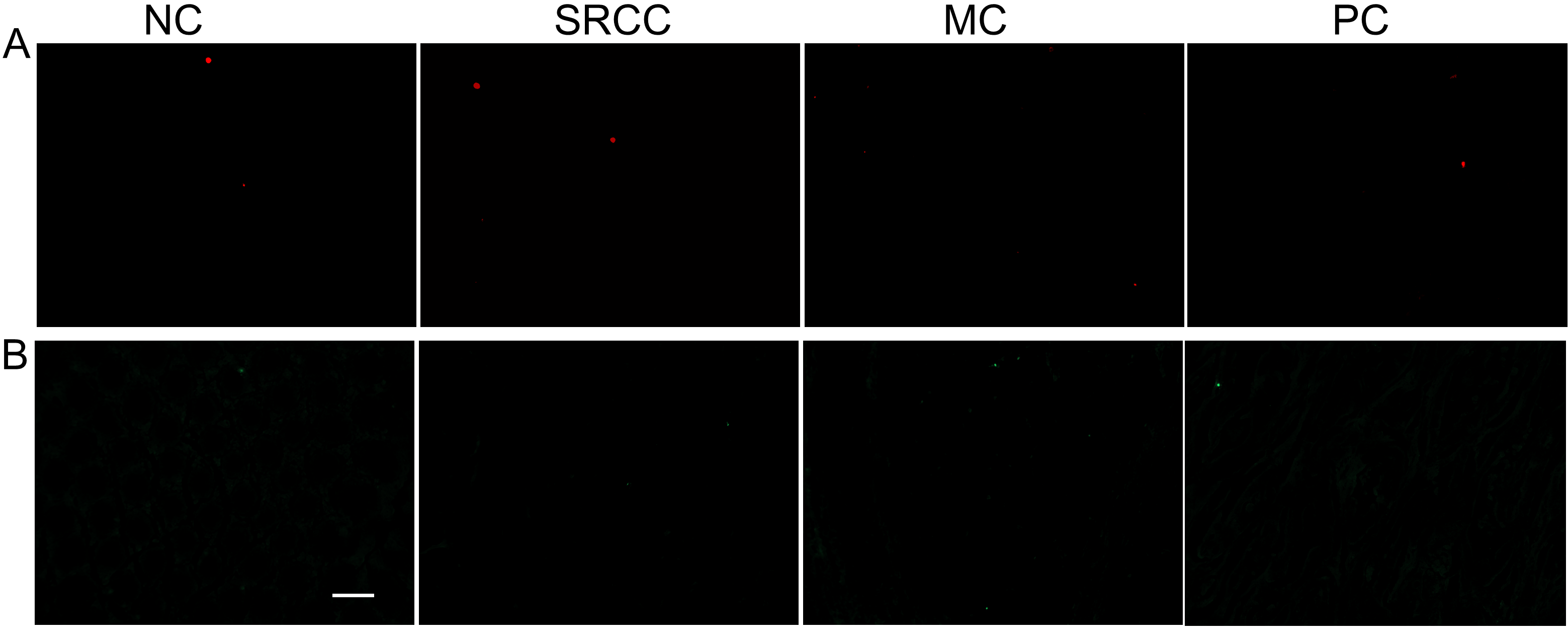

Supplement: S3 Fig — The IF analysis of tissues using isotype controls included (A) rIgM for HECA-452 and (B) hIgG for L-selectin. Imaging conditions for isotype controls were used to control for autofluorescence. Scale bar = 100 μm. Images were acquired using a 10x objective. Tissue sections were cut from FFPE tissue blocks and data are representative of n = 3 independent experiments, as described in Methods. (TIF) [file pone.0173747.s003.tif]

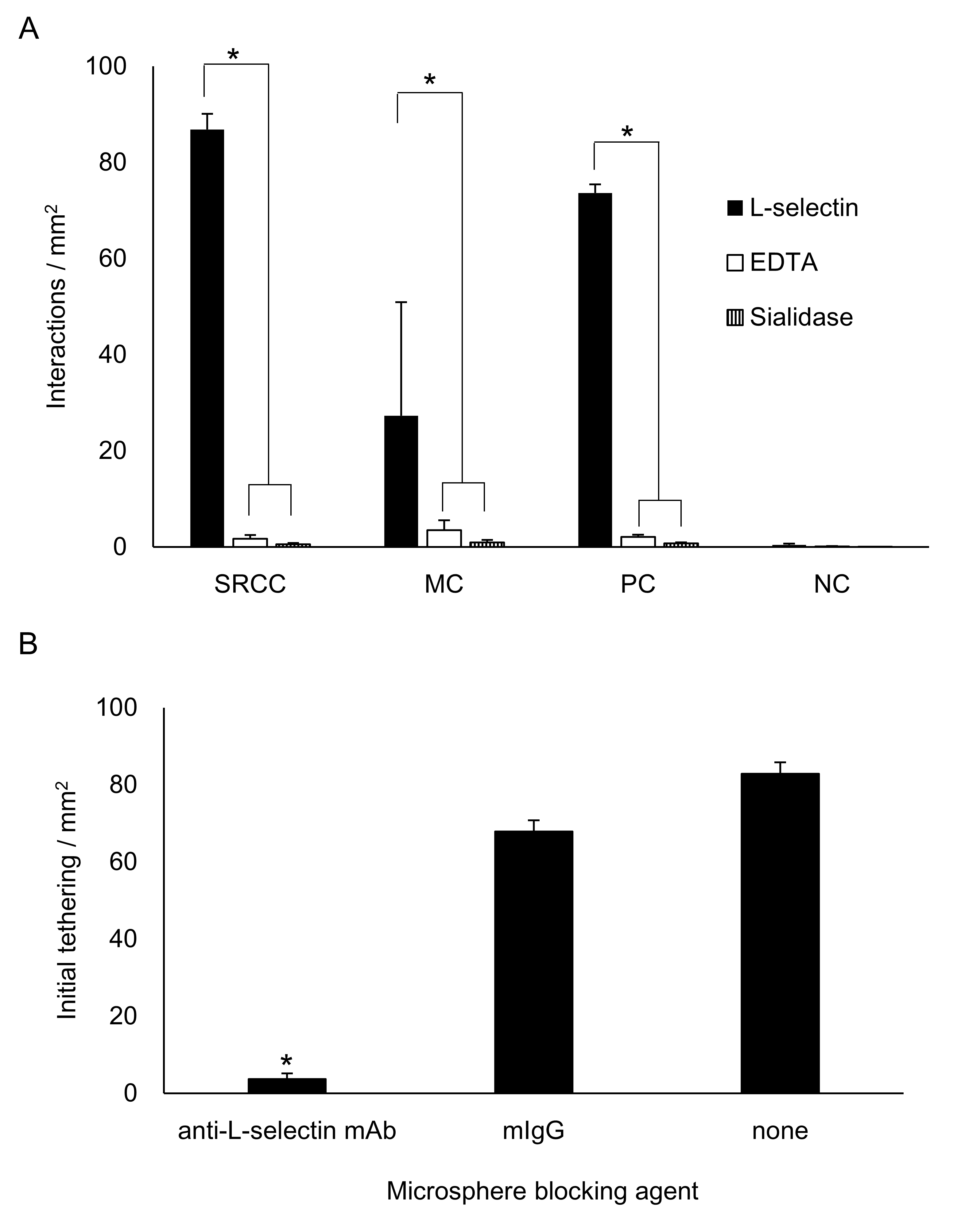

Supplement: S4 Fig — (A) Significantly fewer L-selectin microsphere interactions occurred on colon cancer tissues in the presence of EDTA (5mM) or on colon cancer tissues treated with sialidase. Data are mean ± SEM for n = 3 independent experiments, as described in Methods (*P<0.05). (B) The initial tethering of L-selectin microspheres to SRCC cancer tissue was significantly decreased (almost to complete blockade) by function blocking anti-Lselectin mAb but not mIgG isotype control. Data are mean ± SEM for n = 3 replicate flow assays on one SRCC tissue section (*P<0.05). Tissue sections were cut from FFPE tissue blocks as described in Methods. (TIF) [file pone.0173747.s004.tif]

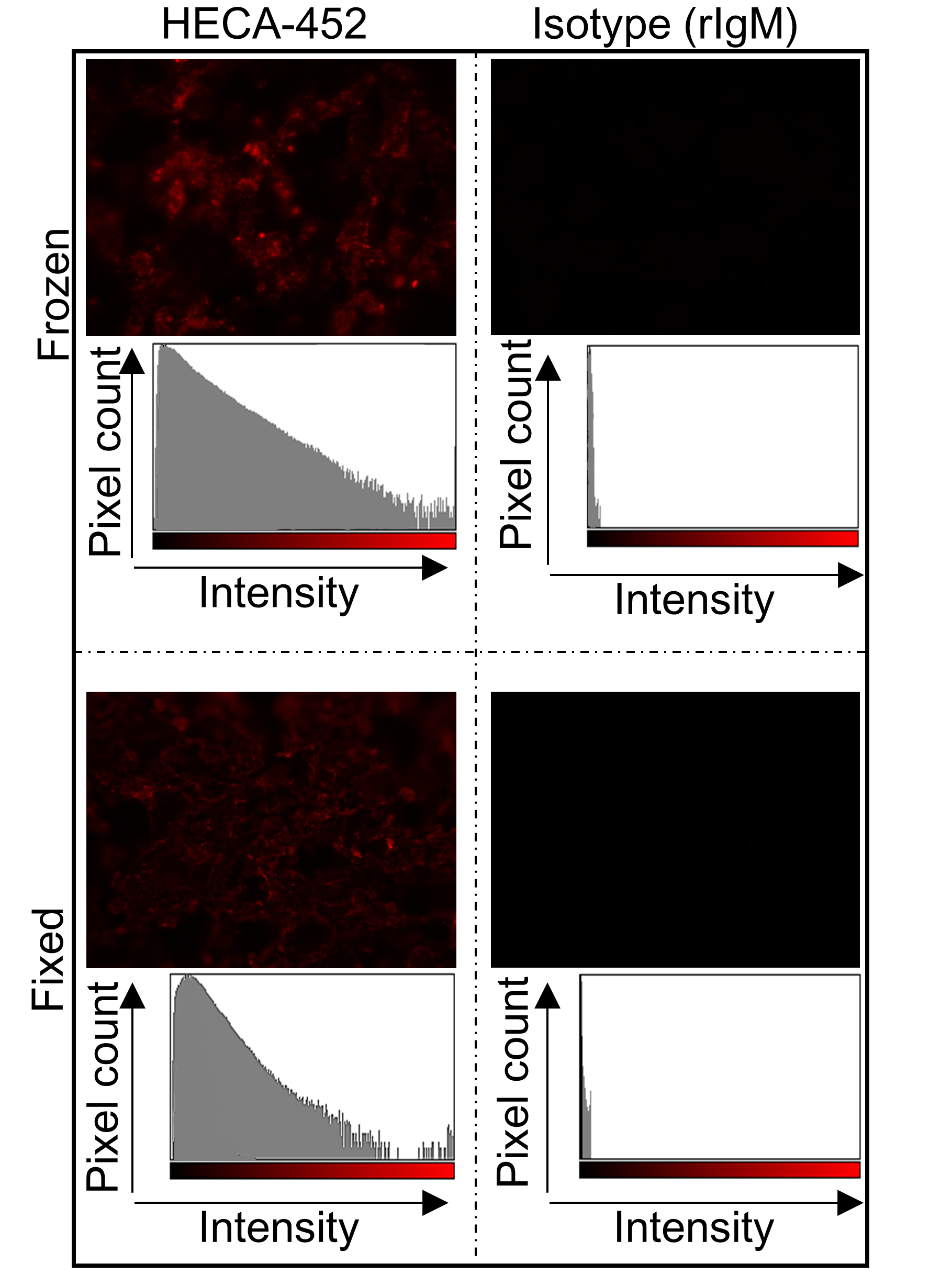

Supplement: S5 Fig — Histograms report the fluorescence intensity of image pixels from IF of frozen or fixed Ls174T tissues assayed with HECA-452 mAb or rIgM isotype control. A greater number of pixels had higher HECA-452 fluorescence intensities on frozen tissue than on fixed tissue, yet tissues stained with HECA-452 had greater intensities than the isotype control tissues regardless of the preparation technique. Data shown are, signal intensities collected from a single representative tissue section of frozen or FFPE tissues (corresponding to n = 3 experiments in Fig 7). Scale bar = 100 μm. (TIF) [file pone.0173747.s005.tif]
